# The Book World of Medicine and Science

**Published:** 1898-01-15

**Authors:** 


### THE BOOK WORLD OF MEDICINE AND SCIENCE.

THE POCKET THERAPIST: A CONCISE MANUAL OF MODERN TREATMENT. By THOS. STRETCH DOWSE, M.D. (Bristol: John Wright and Co. 1897. Price 5s.)

This purports to be an alphabetical guide to the treatment of disease. Evidently the author has had a pretty large personal experience, and perhaps this very fact may have interfered to some extent with the success of his attempt to produce a useful manual of treatment. The personal element prevails throughout, and although the treatment which the author is accustomed to use seems well presented, there is much in the book which is open to question, especially as a guide to practice.

LOCALISATION OF HEADACHE AND SICK HEADACHE, INDICATING THEIR ORIGIN, PATHOLOGY, AND TREATMENT. By H. BENDELACK HEWETSON, F.L.S., &c., Hon. Ophthalmic and Aural Surgeon, Leeds General Infirmary. (London: Simpkin, Marshall, and Co. Leeds: Goodall and Suddick. 1897. 140 pages, 32 diagrams.)

The volume consists of reprints of two papers, one read before the Leeds Medico-Chirurgical Society in 1884, and the other at a meeting of the British Medical Association in 1888—a chapter of miscellaneous remarks, and 32 diagrams. In 1884 Mr. Hewetson, like many other ophthalmic surgeons, became impressed with the fact that headaches were frequently due to the straining of eyes with uncorrected errors of refraction. He was able to record several cases in which, not only the headaches, but also the accompanying dyspepsia and vomiting, were relieved by the use of glasses which corrected the optical defect. In 1888 he became desirous of showing, to use his own words and give an example of his style of writing, "that the neuroses growing and arising from congenital optical error are more subtle in their nature, more varied in their distribution, and more demoralising to the right evolution of the nervous system than I was first led to suppose." He further discovered, by what he describes as "strange good fortune, that in many cases the headaches—or attacks of migraine—were evidently in no way connected with any optical error, or they were only partially so." He was therefore led to extend his researches in these cases to the teeth, and later to the condition of the naso-pharynx. He was then able to assure himself that "quiescent, non-aching teeth" were sometimes the cause of the trouble. Probably at some future date Mr. Hewetson may, in treating individuals with neurotic disturbances, find sources of peripheral irritation which are not confined to the head. Mr. Hewetson's method of recording cases leaves much to be desired; he does not give any description of the patient's general condition, or the state of any organs of the body except the eyes. He contents himself with mentioning the error of refraction, and the result obtained by the use of glasses. He refers to a case of epilepsy cured by the correction of mixed astigmatism, but in such brief terms that the record is practically valueless. An examination of his diagrams would lead us to suppose that he was desirous of establishing a new form of phrenology, so that a patient would only have to locate accurately the position of a pain in the head and the surgeon would know what part was the cause of the trouble, eyes, teeth, or nose, &c. It is to be regretted that Mr. Hewetson, before writing on such a well-worn subject as the connection of eye strain and headache, should not have put himself *au courant* with the recent literature on the subject.

THE SOUTH AFRICAN CLIMATE, INCLUDING CLIMATOLOGY AND BALNEOLOGY, &c. By WILLIAM C. SCHOLTZ, M.P., of Cape Town. Pp. 195. Eleven illustrations. (London, Paris, and Melbourne: Cassell and Co. 1897.)

This interesting volume is written in two sections. The first, from the pen of Dr Scholtz, deals with general considerations; the second, by various writers, is a series of articles on special subjects and localities. The whole forms a book that should be in the hands of every physician who contemplates sending patients to South Africa. Dr. Scholtz and his coadjutors certainly do not under-estimate the remarkable advantages to tuberculous patients accruing from residence in South Africa; but, at the same time, the book is remarkably free from the fulsome and extravagant claims so often put forward in works of this class. Dr. Scholtz is careful to point out the necessity for discrimination in the selection of cases for climatic treatment, and urges the necessity of obtaining local and expert advice on arriving at Cape Town. In England we are apt to forget the many varieties of conditions of life that obtain within the geographical region known as South Africa; and patients are often sent out to die struggling "up country" when they would do better in a sanatorium on the coast. The book is one to be carefully read and studied.

A MANUAL OF URINE TESTING. By JOHN SCOTT, B.A., M.B. Third edition, enlarged. 1897. (Dublin: Fannin and Co. London: Baillière, Tindall, and Cox. Pp. 52, 12mo. Price 1s. 6d.)

This little book is, of its kind, good. That is to say, within a small compass it supplies a vast amount of information clearly arranged and for the most part accurate. It relates not only to testing, but to the clinical importance of the results obtained by testing, and to that end Mr. Hurry Fenwick's tables are made free use of. But the student who cannot learn what he ought from text-books of medicine and practical ward work should not be allowed to cram from little books; and the student who knows his work will not want to.

### BOOKS RECEIVED.

SWAN SONNENSCHEIN AND CO.

"Introduction to the Study of Organic Chemistry." By John Wade, B.Sc., Lond.

ISBISTER AND CO.

"Lincoln Cathedral." By the Rev. Edmund Venables, M.A. Illustrated by Herbert Railton.

BRITISH MEDICAL ASSOCIATION.

